# Level of Burden and Health-Related Quality of Life in Caregivers of Palliative Care Patients

**DOI:** 10.3390/ijerph16234806

**Published:** 2019-11-29

**Authors:** Juana Perpiñá-Galvañ, Núria Orts-Beneito, Manuel Fernández-Alcántara, Sofía García-Sanjuán, María Paz García-Caro, María José Cabañero-Martínez

**Affiliations:** 1 Department of Nursing, University of Alicante, 03690 Alicante, Spain; juana.perpina@ua.es (J.P.-G.); sofia.garcia@ua.es (S.G.-S.); mariajose.cabanero@ua.es (M.J.C.-M.); 2 Institute of Health and Biomedical Research of Alicante (ISABIAL-FISABIO Foundation), 03010 Alicante, Spain; nuriovich@hotmail.com; 3 Department of Health Psychology, University of Alicante, 03690 Alicante, Spain; 4 Department of Nursing, University of Granada, 18016 Granada, Spain; mpazgc@ugr.es

**Keywords:** palliative care, caregiver, burden, anxiety, depression, quality of life

## Abstract

The complexity of palliative care means that the emotional distress and burden that primary family caregivers suffer under can be particularly high. The objective of this study was to determine the level of burden endured by these primary family caregivers and to identify the variables that predict it in the caregiving relatives of people who require home-based palliative care. A descriptive-correlational cross-sectional study was conducted. Socio-demographic and clinical data were collected from caregivers through a self-administered questionnaire that included questions from the 12-Item Short Form Health Survey (SF-12), Zarit Caregiver Burden Interview (ZBI), Hospital Anxiety and Depression Scale (HADS), Brief Resilient Coping Scale (BRCS), Post Traumatic Growth Inventory (PTGI), and Fatigue Assessment Scale (FAS). A total of 77 caregivers participated; 66.2% were women, and the mean age was 61.5 years. Most (62.3%) were providing care to cancer patients. From among these data, the presence of anxiety as a clinical problem (48.1%), a high average fatigue score (FAS) of 23.0 (*SD* = 8.5), and the prevalence of intense overload (41.6%) stood out. We found statistically significant correlations between the variables of burden, fatigue, post-traumatic growth, anxiety, and depression, with the latter two being the main predictive variables of burden. In addition, caregiver burden was associated with a worsening of health. Identifying the factors that influence the appearance of overburden will allow the specific needs of careers to be assessed in order to offer them emotional support within the healthcare environment.

## 1. Introduction

The population-level increase in age and dependency is generating new challenges linked to these new problems and requirements within socio-economic, cultural, and healthcare environments [1,2]. The need for long-term care by older adults with multiple pathologies, dementia, or advanced chronic diseases has meant that ‘primary family caregiver’ (PFC) support has grown in importance. These caregivers who are usually relatives support most of the physical and emotional burdens of care [3,4] without receiving any financial compensation [3,4,5,6,7,8]. The importance of their role not only lies in the fact that they become the main providers of health care, but also because they allow the receivers of care to remain within their social environment; thus, reducing the use of formal resources and delaying or avoiding their admission into care institutions [3].

However, assuming the role of family caregiver and the tasks associated with care generates changes within the family structure and functioning, and is a notable source of stress for caregivers who may assume significant burdens which can negatively impact their own health. The caregiver’s burden has been defined as a multidimensional response to the set of physical, mental, and socioeconomic problems suffered by those providing care to sick people; this disrupts their lives and the consequences can be psychiatric diagnoses, physical disorders, and poor quality of life [9,10,11]. Thus, most studies focus on the negative effects that the caregiver burden has on the health of the caregiver with the appearance of anxiety [12,13,14], depression [12,13,14,15,16], and worsening health-related quality of life (HRQoL) [12,17]. In this sense, burden can be understood as a significant predictor of the caregiver’s quality of life [18,19].

However, other authors suggest that HRQoL can improve even when caregivers report elevated burden, reinforcing the conceptual difference between burden and quality of life [20,21,22]. This phenomenon can be explained by the subjective component of burden, which refers to the way in which the caregiver perceives the tasks of care giving, so that they may feel burden but still value the care as an experience that enriches their life [15,17]. Some research in cancer and dementia patients has indicated that care provision has some positive elements, mainly in terms of family resilience and post-traumatic growth. Family resilience has been linked to a greater capacity to acquire new strengths and greater social resources [23] and has been inversely related to depression, anxiety, and perceived burden [24]. Post-traumatic growth has been linked to a decrease in the feelings of stress, improved family coping, and increased health [25]. 

Considering these contradictory results in the relationship between burden and life quality, it is important to deepen our analysis of the HRQoL variables associated with caregiver burden in different contexts and to analyse the effects of burden on the health of those providing care as part of the holistic and comprehensive approach that characterises end-of-life care [11,14].

Therefore, the objectives of the present research were: (1) to determine the level of burden of the PFCs of people with advanced diseases that require palliative care provided at home and (2) identify the variables that predict these burden levels among these caregivers. In this regard, we expected to find strong significant associations between caregiver burden, depression, and anxiety, along with lower associations with fatigue, post-traumatic growth, resilience, and health-related quality of life measures.

## 2. Materials and Methods 

### 2.1. Study Design and Participants

This was a cross-sectional descriptive study carried out with the relative caregivers of patients receiving palliative care at home and followed-up by case management nurses from seven healthcare centres in the provinces of Alicante and six of the provinces in Granada and Cordoba (Spain). The sample was selected based on intentional non-probabilistic sampling, in accordance with the following inclusion criteria: (a) the person was a primary family caregiver of patient(s) aged over 18 years with advanced oncological or non-oncological pathologies, but without cognitive impairment, requiring palliative care; (b) over 18 years old; and (c) was able to understand and express themselves correctly in Spanish. Formal caregivers who received compensation were excluded. 

### 2.2. Measures 

A data collection questionnaire was prepared that included patient data (age, sex, medical diagnosis, and their subjective perception of the burden that their need for care fulfilled by their main caregiver); the sociodemographic data of the primary caregiver (age, sex, nationality, city of residence, marital status, educational level, employment status, annual family income, and religion and participation in religious rites); care-related information (time the caregiver spent administering care and caregiver overload); caregiver HRQoL; and caregiver psycho-emotional variables (anxiety, depression, resilience, posttraumatic growth, and fatigue) were also collected. 

To avoid the order effects of the instruments, three models of the questionnaire, changing the order of which the instruments were presented, were designed. The following measures were used:

- The 12-Item Short Form Health Survey (SF-12) [26]: Adapted to Spanish by Alonso et al. [27]. This questionnaire assesses HRQoL and is comprised of 12 items of the SF-36 [28]. It is used to define an individuals’ positive and negative state of physical and mental health through eight dimensions (physical function, physical role, body pain, mental health, general health, vitality, social function, and emotional role). There are three to six response options (depending on the item) measured on a Likert scale which evaluate the intensity and/or frequency of health status. The score ranges from 0 to 100, where the highest score implies a better HRQoL [29]. The SF-12 is a valid and reliable metric (α > 0.70) and significant correlations have been shown between its different versions [30,31,32,33].
- The Zarit Caregiver Burden Interview (ZBI) [34]: Created to assess caregiver burden and adapted to Spanish by Martín et al. [35]. This is a multidimensional questionnaire (physical, emotional-psychological, social, and economic) containing 22 items and scored on a five-point Likert scale where 0 = “never” and 5 = “almost always.” The score ranges from 22 to 110 points, classified in the following way: “absence of burden” (≤46), “light burden” (47–55), or “intense burden” (≥56). The ZBI is a widely used and validated test for different populations [36]. It has good internal consistency (α = 0.92) [37], a sensitivity of 98.5%, and a specificity of 93.9% [38].
- Hospital Anxiety and Depression Scale (HADS) [39]: Designed to assess anxiety and depression and adapted to Spanish by Tejero et al. [40]. The HADS contains two subscales, one for anxiety and the other for depression, each comprised of 7 items (resulting in a total of 14 items). Each item is evaluated on a three-point Likert scale on which the higher the score, the greater the anxiety and depression for both subscales. The overall score is classified as: “normal” (0–7 points), “doubtful” (8–10), or a “clinical problem” (>11) [41]. It has been validated and translated into numerous languages (French, Italian, Chinese, German, and Spanish, etc.) and used in various countries and cultures [42,43,44]. The Spanish adaptation has been tested in different populations [45,46,47], has shown good internal consistency both for the anxiety subscale (α = 0.83) and for the depression subscale (α = 0.87) [48], and has shown sensitivity ranges between 0.74 and 0.84 and specificity ranging between 0.78 and 0.80 [49].
- Brief Resilience Coping Scale (BRCS) [50]: Designed to assess resilience in multiple populations. The BRCS was validated in Spanish by Limonero et al. [51] and consists of 4 items with a score obtained on a five-point Likert scale where 1 = “does not describe me at all” and 5 = “describes me very well.” The total score ranges from 4 to 20, with higher scores denoting greater resilience. A total score equal to or less than 13 indicates low resilience, while scores equal to or greater than 17 indicate high resilience. The BRCS has shown adequate internal consistency (α = 0.78) and a test-retest reliability of 0.71 512].
- Post Traumatic Growth Inventory (PTGI) [52]: This questionnaire assesses the perception of personal benefits among survivors of a traumatic event. The Spanish translation of the PTGI, published by Páez et al. [53], was used for this present study. It consists of 10 items and uses a Likert-type response scale with 6 categories ranging from 0 = “no change” to 5 = “very high,” in a positive sense. The scale showed high internal consistency (α = 0.95) and a reliability of 0.95 using the Guttman coefficient.
- Fatigue Assessment Scale (FAS) [54]: This questionnaire evaluates the physical and mental fatigue perceived by the main caregiver and comprises 10 items measured on a five-point Likert response scale varying from 1 = “never” to 5 = “always.” Five elements each reflect the physical and psychological component, respectively. The Spanish translation (FAS-e) of the scale showed adequate reliability (α = 0.80) [55].

### 2.3. Procedure

The sample selection process was carried out by case management nurses in their respective health centres. After obtaining the contact details of a case, a member of our research team contacted the PFC by telephone to request their consent and to arrange an appointment at their preferred location (their home or at the health centre). On the specified day, a trained investigator went to the agreed location to obtain their signed consent document and to provide them with the questionnaire. The caregiver subsequently filled out the questionnaire by themselves in order to provide them with an environment of intimacy and the freedom to respond to the questions in the most reliable way possible. 

This study was part of a larger project jointly developed by the University of Alicante, the Institute of Healthcare, and Biomedical Research of Alicante (ISABIAL), and the University of Granada. The project was authorised by the Health Ethics and Research Committee of the Junta de Andalucía and by the Foundation for the Promotion of Health and Biomedical Research (FISABIO) of the Valencian Community (reference number: PI2017/66). During this work, all the rights of the participants were respected, and they signed their informed consent to participation in writing. All the personal data obtained in this study were treated in accordance with Organic Law 3/2018 of December 5, on the protection of personal data and the guarantee of digital rights as stipulated in Regulation (EU) 2016/679 of the European Parliament and Council, of 27 April 2016 on Data Protection (GDPR).

### 2.4. Data Analysis

A descriptive analysis of the sociodemographic variables and the other main variables (anxiety, depression, fatigue, quality of life, resilience, post-traumatic growth, and burden) was carried out to obtain the means and standard deviations (SDs) for continuous variables and the frequencies and percentages for categorical variables. Because the study sample did not fit a normal curve distribution, contrast and non-parametric analyses were performed. On the one hand, correlations between the main study variables were calculated using Spearman’s rho. On the other hand, differences according to the levels of burden were analysed using Kruskal–Wallis tests. Finally, to understand which quality of life-related variables were predictors of burden levels, we performed a backwards multiple linear regression which included the variables that showed statistically significant associations with caregiver burden. Given the number of comparisons, the level of statistical significance for correlations and group-comparison was set at *p* < 0.007, while for the regression model it was set at *p* < 0.05. The data were analysed using the Statistical Package for the Social Sciences (version 25.0) for Windows (IBM Corp., Armonk, NY, USA).

## 3. Results

### 3.1. Sample Characteristics 

A total of 77 caregivers participated; the majority were women (66.2%) and their mean age was 61.5 years (*SD* = 13.1); 85.7% of the participants were married or living in a partnership and most were retired (35.1%) or were homemakers (27.3%), and had a mid (32.5%) or primary education level (26.45%); 68.8% of the sample indicated that they were Catholic (see Table 1). The participating caregivers took care of patients with a mean age of 73 years (*SD* = 13.2), of whom approximately half were men (51.9%) and who had mostly been diagnosed with cancer (62.3%). 

### 3.2. Description of the Main Variables and Their Associations

As shown in Table 2, for 48.1% of the sample, anxiety was a clinical problem and the overall average obtained on the HADS anxiety scale was 10 (*SD* = 4.5). In contrast, only 18.2% of the participants showed indicators of depression as a clinical problem and the overall mean was 6.6 (*SD* = 4.2). The average score on the FAS scale was 23.0 (*SD* = 8.5) and almost half of the participants indicated experiencing substantial fatigue (48.1%). The mean scores for the physical and mental components of the HRQoL were similar at 41.2 (*SD* = 5.9) and 41.5 (*SD* = 9.7), respectively. The participants’ resilience levels were mostly low (48.1%) or in the normal range (32.5%), with an average of 13.9 (*SD* = 3.1). The average post-traumatic growth score obtained by the participating caregivers was 27.4 (*SD* = 13.1). Finally, in relation to the main study variable, 63.7% of the sample showed burden, with 22.1% of individuals being mildly burdened and 41.6% of individuals being intensely burdened. The mean ZBI score was 52.2 (*SD* = 16.1). 

As shown in Table 3, the anxiety, depression, fatigue, post-traumatic growth, and caregiver burden showed significant correlations between each other. Associations with burden ranged between 0.36 (*p* = 0.001) with post-traumatic growth and 0.70 (*p* < 0.001) with depression. Neither the physical and metal components of HRQoL, nor resilience, showed any significant relationships with the rest of the variables. 

### 3.3. Differences in the Variables Related to Health-Related Quality of Life Depending on the Levels of Burden

Comparisons made according to the degree of burden (no burden, slight burden, or intense burden), showed significant differences between the groups for the anxiety, H(2) = 28.70, *p* < 0.001; depression, H(2) = 36.40, *p* < 0.001; post-traumatic growth, H(2) = 10.55, *p* = 0.005; and fatigue H(2) = 18.81, *p* < 0.001 variables (see Table 4). For the variables of anxiety, depression, and fatigue, post-hoc comparisons showed statistically significant differences (*p* < 0.007) between the levels of non-burden and the two remaining categories: mild burden and intense burden (see Table 4). 

### 3.4. Predictive Model of Caregiver Burden

To calculate the predictive model of caregiver burden, only factors that were significantly associated with the outcome variable in the bivariate analysis (anxiety, depression, fatigue, and post-traumatic growth) were selected and were introduced into the model as predictive variables. The “backward” multiple linear regression model showed that depression and anxiety were the main predictive variables. Post-traumatic growth remained in the model, but had less predictive power and no statistical significance, while fatigue was excluded from the model (see Table 5). R^2^ in the model was 52.4, predicting half of the variability in the dependent variable of caregiver burden. 

## 4. Discussion

The objectives of this study were to determine the level of burden of the PFCs of people with advanced diseases that require palliative care provided at home, and to identify the variables that predict these burden levels among these caregivers.

In the results, we describe the profiles and defining features of primary family caregivers for these types of patients and the prevalence of their emotional problems, relating the latter to HRQoL and other variables that best predict burden in them. 

First, with respect to the caregiver profiles, the sample used in this present study presented sociodemographic characteristics similar to those described in previous research [3,8]: they were predominantly women (66%), aged an average of 61 years, who were mostly Catholic, retired, or homemakers, with a basic or medium level of education, living as a couple while administering care. On the other hand, coinciding with the details in other investigations [22], half of the patients were men, their mean age was 73 years, and the diagnosis of cancer predominated.

Secondly, by analysing the cut-off points and categories available for the variables, the present study identified that high levels of anxiety, depression, and fatigue also appeared along with the phenomenon of burden in the case of palliative care caregivers. Higher rates of anxiety than of depression (reaching values of up to 43% and 40%, respectively) were described in previous studies [8,18,22,56]. Similarly, 48% of caregivers in our cohort also reported anxiety as a clinical problem. 

However, there were greater differences with respect to depression in our study, with depression only present in 18% of the sample compared to 39% in the research by Rodrigues-Gomes [5] or 41% in the study carried out by Ullrich et al. [8]. Some research has indicated that there is a relationship between the context of care and the perceived burden. In this sense, a possible explanation is that in our cultural context, in which the family structure is a basic pillar, there is more social support perceived by caregivers, and that helps them not to experience some negative psychological effects, which include depression [11]. Regarding the level of fatigue, our results also coincide with previous studies which found high levels of fatigue in this type of caregiver [12,57]. In terms of the positive elements of care, our findings regarding resilience coincide with other research in which the lower the resilience became, the higher the level of perceived burden was present [23,58,59]. The results we obtained for the PTGI [51] were similar to those reported in studies conducted with PFCs both of palliative [60] and non-palliative patients [23], coinciding with the finding that post-traumatic growth increases regardless of the burden. 

All the elements analysed above coexist, and in turn, form part of the same burden syndrome. The prevalence of burden found in our sample was similar to that reported by other studies, such as González-Guerra [61] conducted with people with palliative needs. However, when compared with studies with populations receiving non-palliative care (mainly the PFCs of patients with dementia), we found a higher prevalence of burden [4,18,19,62,63]. This finding may be related to the chronic, degenerative, and disabling nature of these diseases, whose evolution until reaching the final disease stages can continue for decades. This means that all the negative effects derived from care (e.g., anxiety and depression [58]; distress, stress, fatigue, and insomnia [7,62]; and social isolation, lack of free time, and a deteriorating economic situation [3,4]) are present for a longer time among these PFCs, increasing the risk of worsening different dimensions of their HRQoL. However, our level of burden was higher than that found in a comparative study in caregivers of patients with advanced cancer, dementia, and brain injuries [64]. In addition, a caregiver’s age could be influencing the level of perceived burden, since it has been suggested that caregivers of younger spouses are significantly more engaged than caregivers of older spouses [65]. 

Third, in line with previous studies [12,13,14,25], the strong association between levels of burden and anxiety, depression, fatigue, and PTG was evident in our analysis of the associations between the different variables. However, the fact that neither QoL nor resilience had statistically significant relationships with the rest of the variables contradicts other studies which state that resilience is not only related to burden but also predicts lower burden levels [58] or that resilience has positive effects on the patient’s quality of life but not on the perception of caregiver burden [34]. Nevertheless, our findings are consistent with Krug et al.’s [66] findings in which there was no association between the decrease in overall patient quality of life and an increase in caregiver burden [66]. In addition, a recent longitudinal study did not find a relationship between patients’ quality of life and caregivers’ well-being, which was rather influenced by caregiver engagement [67]. The low association between caregiver burden and other patient variables (i.e., physical functioning and pain) might be due to coping strategies of family caregivers which help them to regulate their emotions. Considering this fact, the low association between quality of life and resilience with the burden perceived by the caregiver may also be related to other patient variables, such as physical functioning and pain. These variables together with the different coping strategies of family caregivers can cause caregivers to subjectively not perceive care as a burden. 

Finally, our linear regression model explained a large percentage of the burden variance, especially in terms of the role of depression and anxiety. This is in line with previous studies in which psychological distress, characterised by the presence of high levels of anxiety and depression, was shown to be directly related to the existence of caregiver burden. Thus, it seems that both these variables can currently predict the development of burden syndrome in PFCs [8,68,69].

These results have important clinical implications. According to Bekdemir and Ilhan [70], the caregiver’s burden is associated with an increase in the severity of the physical, mental, and social problems that caregivers suffer as a result of performing the task of caring. Knowing the factors that influence the appearance of such burden in PFCs providing palliative care to people in need, will allow their specific needs to be assessed in order to determine the support services that caregivers could be offered by healthcare professionals [14]. This support could translate into an improvement in the competence and self-esteem of caregivers, increased post-traumatic growth, better patient-caregiver relationships, and a reduction in anxiety and depression levels and in perceived burden [15]; additionally, a general increase in PFCs’ quality of life and the quality of care they provide [8,14]. Based on our findings, more research is needed to deeply study the factors that influence caregiver overload in order to improve the care they receive during this process. It is important to explore the differences in the perceived overload according to the patient’s diagnosis, and the influence of coping strategies and their relationship with caregiver’s functionality.

This work had a number of limitations. First, given its cross-sectional design, we could not establish any causal relationships between the variables, nor could we know if the levels of anxiety, depression, or fatigue were high before the caregiver began their work. Second, because of our specific study population (caregivers providing care to people at the end of their lives), it was difficult to access the sample, because more elements can delay or prevent participation at this disease stage, including the high demand for care required by patients at home or repeated patient hospital admissions. Finally, the sample size in the present study was relatively small, which may limit the generalizability of the results. It is possible that some relationships between variables were undetected due to this limitation, so future studies with larger samples are needed.

## 5. Conclusions

In conclusion, the present study shows the characteristics of a population of caregivers of palliative care patients, and the relationship between burden, anxiety, depression, fatigue, and PTG in this population. Anxiety and depression seem to be the variables that most predict burden levels in caregivers of patients under palliative care.

## Figures and Tables

**Table 1:** Sample characteristics (*n* = 77).

Variables	% (*n*)
**Sex**	
Male	33.8 (26)
Female	66.2 (51)
**Age (Mean [*SD*])**	61.5 (13.1)
**Civil status**	
Single	6.5 (5)
Married or lives with a partner	85.7 (66)
Widowed or divorced/separated	7.8 (6)
**Employment status**	
Part-time employee	9.1 (7)
Full-time employee	18.2 (14)
Unemployed	10.4 (8)
Retired	35.1 (27)
Homemaker	27.3 (21)
**Education level**	
No formal education	19.5 (15)
Primary education	26.4 (28)
Mid-level education	32.5 (25)
Higher education	11.7 (9)
**Religion**	
Catholic	68.8 (53)
Jehovah’s Witness	1.3 (1)
Agnostic/Atheist	29.9 (23)
**Patient age (Mean [*SD*])**	73.0 (13.2)
**Patient sex**	
Male	48.1 (37)
Female	51.9 (40)
**Patient diagnosis**	
Cancer	64.9 (50)
Organ insufficiencies	19.5 (15)
Neurodegenerative diseases	16.8 (13)

**Table 2:** Description of the main variables.

Variables	% (*n*)	Mean (*SD*)
**Anxiety**		
Normal	28.6 (22)	
Doubtful	23.4 (18)	
Clinical problem	48.1 (37)	10.0 (4.5)
**Depression**		
Normal	59.7 (46)	
Doubtful	22.1 (17)	
Clinical problem	18.2 (14)	6.6 (4.2)
**Fatigue**		
No fatigue	51.9 (40)	
Substantial fatigue	48.1 (37)	23.0 (8.5)
**Quality of life**		
Physical		41.2 (5.9)
Emotional		41.5 (9.7)
**Resilience**		
Low	46.8 (36)	
Normal	32.5 (25)	
High	20.8 (16)	13.9 (3.1)
**Post-traumatic growth**		27.4 (13.1)
**Burden**		
No burden	36.4 (28)	
Slight burden	22.1 (17)	
Intense burden	41.6 (32)	52.2 (16.1)

**Table 3:** Correlations (Spearman rho) between the main variables.

	Anxiety	Depression	Fatigue	Physical QoL	Mental QoL	Resilience	PTG	Burden
Anxiety	1							
Depression	0.701 ***	1						
Fatigue	0.631 ***	0.665 ***	1					
Physical QoL	−0.066	−0.016	−0.015	1				
Mental QoL	−0.086	−0.195	−0.054	0.051	1			
Resilience	−0.088	−0.220	−0.212	−0.166	−0.026	1		
PTG	0.437 ***	0.244 *	0.241 *	−0.154	−0.157	0.151	1	
Burden	0.656 ***	0.700 ***	0.569 ***	−0.047	−0.159	−0.160	0.360 **	1

* *p* < 0.05; ** *p* < 0.01; *** *p* < 0.001 PTG: post-traumatic growth; QoL: quality of life.

**Table 4:** Variable distributions according to the burden levels (*n* = 77).

Variables	Caregiver Burden Categories	*n*	Mean (*SD*)	Post-Hoc
Anxiety	No burden (<47 points)	28	6.6 (3.8)	NB > IB
	Mild burden (47–55 points)	17	10.6 (3.8)	
	Intense burden (>55 points)	32	12.7 (3.4)	
Depression	No burden (<47 points)	28	3.1 (2.3)	NB > MB
	Mild burden (47–55 points)	17	6.8 (3.1)	NB > IB
	Intense burden (>55 points)	32	9.4 (3.7)	
Fatigue	No burden (<47 points)	28	18.1 (6.3)	NB > IB
	Mild burden (47–55 points)	17	23.1 (6.8)	
	Intense burden (>55 points)	32	27.2 (8.9)	
Physical QoL	No burden (<47 points)	28	42.0 (5.2)	
	Mild burden (47–55 points)	17	39.2 (6.9)	
	Intense burden (>55 points)	32	41.2 (5.9)	
Mental QoL	No burden (<47 points)	28	43.7 (9.0)	
	Mild burden (47–55 points)	17	41.7 (8.5)	
	Intense burden (>55 points)	32	39.1 (10.3)	
Resilience	No burden (<47 points)	28	14.0 (3.2)	
	Mild burden (47–55 points)	17	15.0 (2.7)	
	Intense burden (>55 points)	32	13.2 (3.2)	
PTG	No burden (<47 points)	28	21.1 (13.3)	
	Mild burden (47–55 points)	17	30.6 (14.9)	
	Intense burden (>55 points)	32	31.1 (9.8)	

PTG: post-traumatic growth; NB: no burden; MB: mild burden; IB: intense burden.

**Table 5:** Multiple linear regression model of burden and associated variables.

Variables Included	β	*p*-Value	95% CI
Anxiety	0.266	0.032	0.086–1.823
Depression	0.459	0.000	0.916–2.630
PTG	0.152	0.094	−0.033–0.409

PTG: post-traumatic growth. R^2^ = 0.524.

## References

[1] Abades Porcel M., Rayón Valpuesta E. (2012). El envejecimiento en España: ¿Un reto o problema social?. Gerokomos.

[2] Gómez-Batiste X., Blay C., Roca J., Fontanals M.D. (2012). Innovaciones conceptuales e iniciativas de mejora en la atención paliativa del siglo XXI. Med. Paliat..

[3] López Gil M.J., Orueta Sánchez R., Gómez-Caro S., Sánchez Oropesa A., Carmona de la Morena J., Alonso Moreno F.J. (2009). El rol de Cuidador de personas dependientes y sus repercusiones sobre su Calidad de Vida y su Salud. Rev. Clín. Med. Fam.

[4] Montero Pardo X., Jurado Cárdenas S., Méndez Venegas J. (2015). Variables que predicen la aparición de sobrecarga en cuidadores primarios informales de niños con cáncer. Psicooncología.

[5] Rodrigues Gomes A.M. (2010). El cuidador y el enfermo en el final de la vida—Familia y/o persona significativa. Enferm. Glob..

[6] Espinoza Miranda K., Jofre Aravena V. (2012). Sobrecarga, apoyo social y autocuidado en cuidadores informales. Cienc. Enferm..

[7] Lund L., Ross L., Petersen M.A., Groenvold M. (2014). Cancer caregiving tasks and consequences and their associations with caregiver status and the caregiver’s relationship to the patient: A survey. BMC Cancer.

[8] Ullrich A., Ascherfeld L., Marx G., Bokemeyer C., Bergelt C., Oechsle K. (2017). Quality of life, psychological burden, needs, and satisfaction during specialized inpatient palliative care in family caregivers of advanced cancer patients. BMC Palliat. Care.

[9] Tripodoro V., Veloso V., Llanos V. (2015). Sobrecarga del cuidador principal de pacientes en cuidados paliativos. Publicación del Instituto de Investigaciones Gino Germani. Facultad de Ciencias Sociales.

[10] Breinbauer H., Vásquez H., Mayanz S., Guerra C., Millán T. (2009). Validación en Chile de la Escala de Sobrecarga del Cuidador de Zarit en sus versiones original y abreviada. Rev. Méd. Chile.

[11] Choi S., Seo J. (2019). Analysis of caregiver burden in palliative care: An integrated review. Nurs. Forum.

[12] Lai C., Luciani M., Di Mario C., Galli F., Morelli E., Ginobbi P., Lombardo L. (2017). Psychological impairments burden and spirituality in caregivers of terminally ill cancer patients. Eur. J. Cancer Care.

[13] Strang V.R., Koop P.M., Peden J. (2002). The experience of respite during home-based family caregiving for persons with advanced cancer. J. Palliat. Care.

[14] Zavagli V., Raccichini M., Ercolani G., Franchini L., Varani S., Pannuti R. (2019). Care for carers: An investigation on family caregivers’ needs, tasks, and experiences. Transl. Med. Unisa.

[15] Petursdottir A.B., Svavarsdottir E.K. (2019). The effectivness of a strengths-oriented therapeutic conversation intervention on perceived support, well-being and burden among family caregivers in palliative home-care. J Adv Nurs..

[16] Sherman D.W. (1998). Reciprocal suering: The need to improve family caregivers’ quality of life through palliative care. J. Palliat. Care Med..

[17] Stajduhar K.I. (2013). Burdens of family caregiving at the end of life. Clin. Inestig. Med..

[18] Schulz R., Belle S.H., Czaja S.J., Ginnis K.A., Stevens A., Zhang S. (2004). Long-term care placement of dementia patients and caregiver health and well-being. JAMA.

[19] Brodaty H., Donkin M. (2009). Family caregivers of people with dementia. Dialogues Clin. Neurosci..

[20] Argimon J.M., Limón E., Abós T. (2003). Sobrecarga y calidad de vida de los cuidadores informales de pacientes discapacitados. Aten. Primaria.

[21] Chappell N.L., Reid C. (2002). Burden and well-being among caregivers: Examining the distinction. Gerontologist.

[22] Li Y., Qiao Y., Luan X., Li S., Wang K. (2019). Family resilience and psychological well-being among Chinese breast cancer survivors and their caregivers. Eur. J. Cancer Care.

[23] Petriwskyj A., Parker D., O’ Dwyer S., Moyle W., Nucifora N. (2016). Interventions to build resilience in family caregivers of people living with dementia: A comprehensive systematic review. Joanna Briggs Inst..

[24] Behzadi M., Rassouli M., Khanali L., Pourhoseingholi M.A., Alaie F. (2018). Posttraumatic growth and its dimensions in the mothers of children with cancer. Int. J. Community Based Nurs. Midwifery.

[25] Ware J.E., Kosinski M., Keller S.D. (1996). A 12-item short-form health survey: Construction of scales and preliminary tests of reliability and validity. Med. Care.

[26] López-García E., Banegas J.R., Pérez-Regadera A.G., Gutiérrez-Fisac J.L., Alonso J., Rodríguez-Artalejo F. (2003). Valores poblacionales de referencia de la versión española del Cuestionario de Salud SF-36 en población adulta de más de 60 años. Med. Clin..

[27] Ware J.E., Kosinski M., Keller S.D. (1994). SF-36 Physical and Mental Health Summary Scales: A User’s Manual.

[28] Vera Villarroel P., Silva J., Celis Atenas K., Pavez P. (2014). Evaluación del cuestionario SF-12: Verificación de la utilidad de la escala salud mental. Rev. Med. Chile.

[29] Vilagut G., Valderas J.M., Ferrer M., Garín O., López-García E., Alonso J. (2008). Interpretación de los cuestionarios de salud SF-36 y SF-12 en España. Componentes físico y mental. Med. Clin..

[30] Andrews G. (2002). A brief integer scorer for the SF-12: Validity of the brief scorer in Australian community and clinic settings. Aust. N. Z. J. Public Health.

[31] Jenkinson C., Layte R. (1997). Development and testing of the UK SF-12 (short form health survey). J. Health Serv. Res. Policy.

[32] Okonkwo O.C., Roth D.L., Pulley L.V., Howard G. (2010). Confirmatory factor analysis of the validity of the SF-12 for persons with and without a history of stroke. Qual. Life Res..

[33] Zarit S.H., Reever K.E., Bach-Peterson J. (1980). Relatives of the impaired elderly: Correlates of feeling of burden. Gerontologist.

[34] Martín M., Salvadó I., Nadal S., Miji L.C., Rico J.M., Lanz P., Taussing M.I. (1996). Adaptación para nuestro medio de la Escala de Sobrecarga del Cuidador (Caregiver Burden Interview) de Zarit. Rev. Esp. Geriatr. Gerontol..

[35] Crespo M., Rivas M.T. (2015). La evaluación de la carga del cuidador: Una revisión más allá de la escala de Zarit. Clínica y Salud.

[36] Martín-Carrasco M., Otermin P., Pérez-Camo V., Pujol J., Agüera L., Martín M., Gobart A., Pons S., Balana M. (2010). EDUCA study: Psychometric proprieties of the Spanish version of the Zarit caregiver Burden Scale. Aging Ment. Health.

[37] Gort A., March J., Gómez X., De Miguel M., Mazarico S., Ballesté J. (2005). Escala de Zarit reducida en cuidados paliativos. Rev. Med. Clin..

[38] Zigmond A.S., Snaith R.P. (1983). The Hospital Anxiety and Depression Scale. Acta Psychiatr. Scand.

[39] Terol-Cantero M.C., Cabrera-Perona V., Martín-Aragón M. (2015). Revisión de estudios de la Escala de Ansiedad y Depresión Hospitalaria (HAD) en muestras españolas. Anal. Psichol..

[40] Cabrera V., Martín Aragón M., Terol M.C., Núñez R., Pastor M.A. (2015). La Escala de Ansiedad y Depresión Hospitalaria (HAD) en fibromialgia: Análisis de sensibilidad y especificidad. Terapia Psicológica.

[41] Abiodun O.A. (1994). A Validity Study of the Hospital Anxiety and depres-sion scale in general hospital units and a community sample in Nigeria. Br. J. Psychiatry.

[42] Nortvedt M.W., Riise T., Sanne B. (2006). Are men more depressed than women in Norway? Validity of the hospital anxiety and depression scale. J. Psychosom. Res..

[43] Untas A., Aguirrezabal M., Chauveau P., Leguen E., Combe C., Rascle N. (2009). Anxiety and depression in hemodialysis: Validation of the Hospital Anxiety and Depression Scale (HADS). Nephrol Ther..

[44] Terol M.C., López-Roig S., Rodríguez-Marín J., Martín-Aragón M., Pas-Tor M.A., Reig M.T. (2007). Propiedades psicométricas de la Escala Hospitalaria de Ansiedad y Estrés (HAD) en población española. Ansiedad Y Estrés.

[45] Costa Requena G., Pérez Martín X., Salamero Baró M., Gil Moncayo F.L. (2009). Discriminación del malestar emocional en pacientes oncológicos utilizando la Escala de Ansiedad y Depresión Hospitalaria (HADS). Dialnet Plus.

[46] Noguera A., Centeno C., Carvajal A., Portela M.A., Urdiroz J., Martinez M. (2009). Spanish “Fine tuning” of language to describe De-pression and anxiety. J. Palliat. Med..

[47] Vallejo M.A., Rivera J., Esteve Vives J., Rodríguez Muñoz M.F. (2012). Uso del cuestionario Hospital Anxiety and Depression Scale (HADS) para evaluar la ansiedad y la depresión en pacientes con fibromialgia. Rev. Psiquiatr. Salud Ment..

[48] Galindo Vázquez O., Meneses García A., Herrera Gómez A., Caballero Tinoco M.R., Aguilar Ponce J.L. (2015). Escala hospitalaria de ansiedad y depresión (HADS) en cuidadores primarios informales de pacientes con cáncer: Propiedades Psicométricas. Psicooncología.

[49] Sinclair V.G., Wallston K.A. (2004). The development and psychometric evaluation of the brief resilient coping scale. Assessment.

[50] Limonero J.T., Tomás-Sábado J., Gómez-Romero M.J., Maté-Méndez J., Sinclair V.G., Wallston K.A., Gómez-Benito J. (2014). Evidence for validity of the brief resilient coping scale in a young spanish sample. Span. J. Psychol..

[51] Stephen J., Linley A., Harris G.J. (2004). Understanding positive change following trauma and adversity: Structural clarification. J. Loss Trauma.

[52] Páez D., Vázquez C., Bosco S., Gasparre A., Iraurgi I., Sezibera V. (2011). Crecimiento post estrés y post trauma: Posibles aspectos positivos y beneficiosos de la respuesta a los hechos traumáticos. Superando la Violencia Colectiva y Construyendo una Cultura de paz.

[53] Michielsen H.J., De Vries J., Van Heck G.L. (2003). Psychometric qualities of a brief selfrated fatigue measure: The Fatigue Assessment Scale. J. Psychosom. Res..

[54] Cano-Climent A., Oliver-Roig A., Cabrero-García J., De Vries J., Richart-Martínez M. (2017). The Spanish version of the Fatigue Assessment Scale: Reliability and validity assessment in postpartum women. PeerJ.

[55] Hudson P., Payne S. (2011). Family caregivers and palliative care: Current status and agenda for the future. J. Palliat. Med..

[56] Clark M.M., Atherton P.J., Lapid M.I., Rausch S.M., Frost M.H., Cheville A.L., Hanson J.M., Garces Y.I., Brown P.D., Sloan J.A. (2014). Cancer caregiver fatigue: A high level of symptom burden. Am. J. Hosp. Palliat. Care.

[57] Tan J.Y., Molassiotis A., Lloyd-Williams M., Yorke J. (2018). Burden, emotional distress and quality of life among informal caregivers of lung cancer patients: An exploratory study. Eur. J. Cancer Care.

[58] Abdul Z.A., Mccarthy G. (2017). Caregiver burden among caregivers of individuals with severe mental illness: Testing the moderation and mediation models of resilience. Arch. Psychiatr. Nurs..

[59] Navarro-Abal Y., López-López M.J., Climent-Rodríguez J.A., Gómez-Salgado J. (2019). Sobrecarga, empatía y resiliencia en cuidadores de personas dependientes. Gac Sanit..

[60] Hirooka K., Fukahori H., Taku K., Togari T., Ogawa A. (2018). Examining posttraumatic growth among bereaved family members of patients with cancer who received palliative care at home. Am. J. Hosp. Palliat. Med..

[61] González-Guerra A., Fonseca-Fernández M., Valladares-González A., López-Angulo L. (2017). Factores moduladores de resiliencia y sobrecarga en cuidadores principales de pacientes oncológicos avanzados. Rev. Finlay.

[62] Bleijlevens M.H.C., Stolt M., Stephan A., Zabalegui A., Saks K., Sutcliffe C., Lethin C., Soto M.E., Zwakhalen S. (2015). Changes in caregiver burden and health-related quality of life of informal caregivers of older people with Dementia: Evidence from the European Right Time Place Care prospective cohort study. J. Adv. Nurs..

[63] Dueñas E., Martínez M.A., Morales B., Muñoz C., Viáfara A.S., Herrera J.A. (2006). Síndrome del cuidador de adultos mayores discapacitados y sus implicaciones psicosociales. Colomb. Med..

[64] Harding R., Gao W., Jackson D., Pearson C., Murray J., Higginson I.J. (2015). Comparative analysis of informal caregiver burden in advanced cancer, dementia, and acquired brain injury. J. Pain Symptom Manag..

[65] Clipp E.C., George L.K. (1993). Dementia and cancer: A comparison of spouse caregivers. Gerontologist.

[66] Krug K., Miksch A., Peters-Klimm F., Engeser P., Szecsenyi J. (2016). Correlation between patient quality of life in palliative care and burden of their family caregivers: A prospective observational cohort study. BMC Palliat. Care.

[67] Butow P.N., Price M.A., Bell M.L., Webb P.M., DeFazio A. (2014). Caring for women with ovarian cancer in the last year of life: A longitudinal study of caregiver quality of life, distress and unmet needs. Gynecol. Oncol..

[68] Moreira de Souza R., Turrini R.N.T. (2011). Paciente oncológico terminal: Sobrecarga del cuidador. Enfermería Glob..

[69] Grunfeld E., Coyle D., Timothy W., Clinch J., Revno L., Earle C.C., Willan A., Viola R., Coristine M., Janz T. (2004). Family caregiver burden: Results of a longitudinal study of breast cancer patients and their principal caregivers. CMAJ.

[70] Bekdemir A., Ilhan N. (2019). Predictors of caregiver burden in caregivers of bedridden patients. J. Nurs. Res..

